# Supplementary figures and images for: In vivo Host-Pathogen Interaction as Revealed by Global Proteomic Profiling of Zebrafish Larvae
Source: Front Cell Infect Microbiol. 2017 Jul 25;7:334. doi: 10.3389/fcimb.2017.00334 (PMC5524664; doi:10.3389/fcimb.2017.00334)

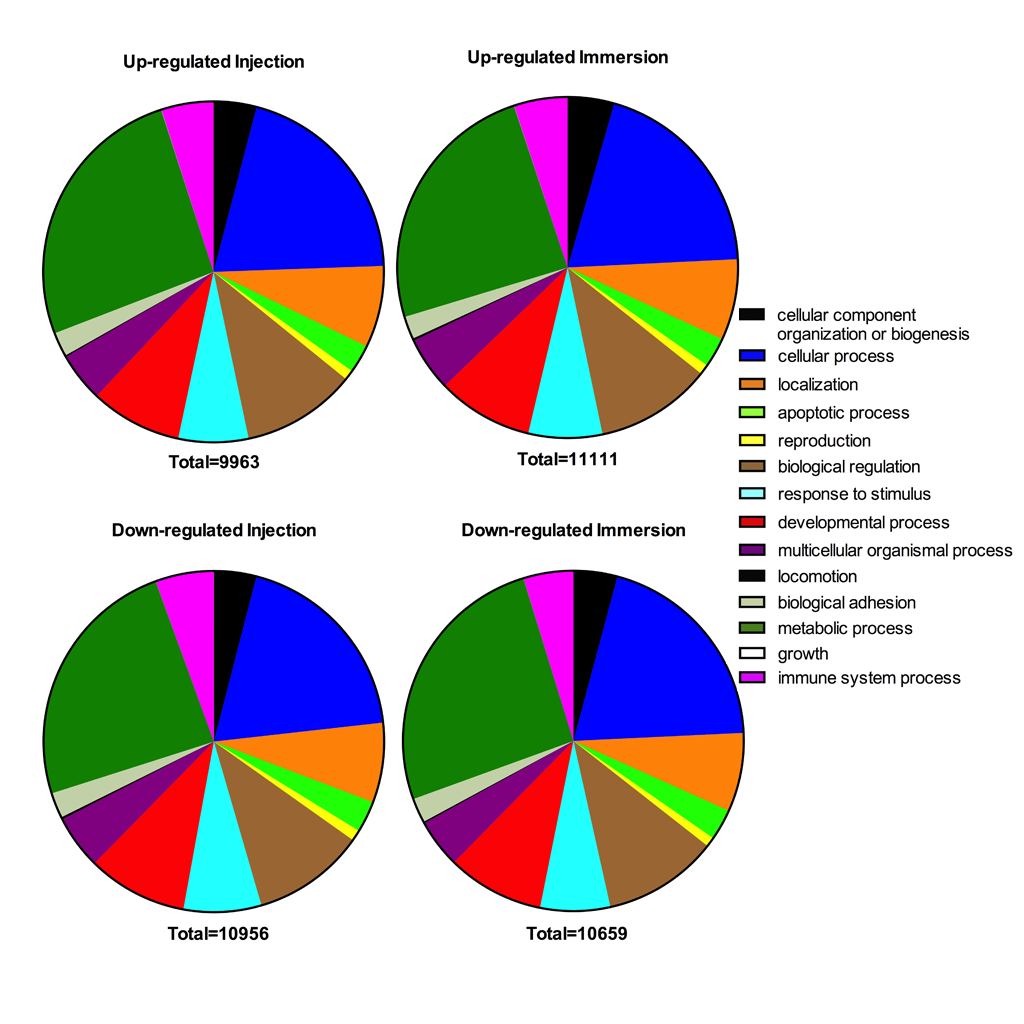

Supplement: Figure S1 — GO annotation of up-regulated and down-regulated proteins in zebrafish exposed to P. aeruginosa by injection or static immersion. Proteins significantly changed at 22 hpe in zebrafish exposed at 72 HPF by injection or static immersion with P. aeruginosa PAO1 were categorized by biological process (GO). The total represents the total matches in the database. [file Image1.TIF]

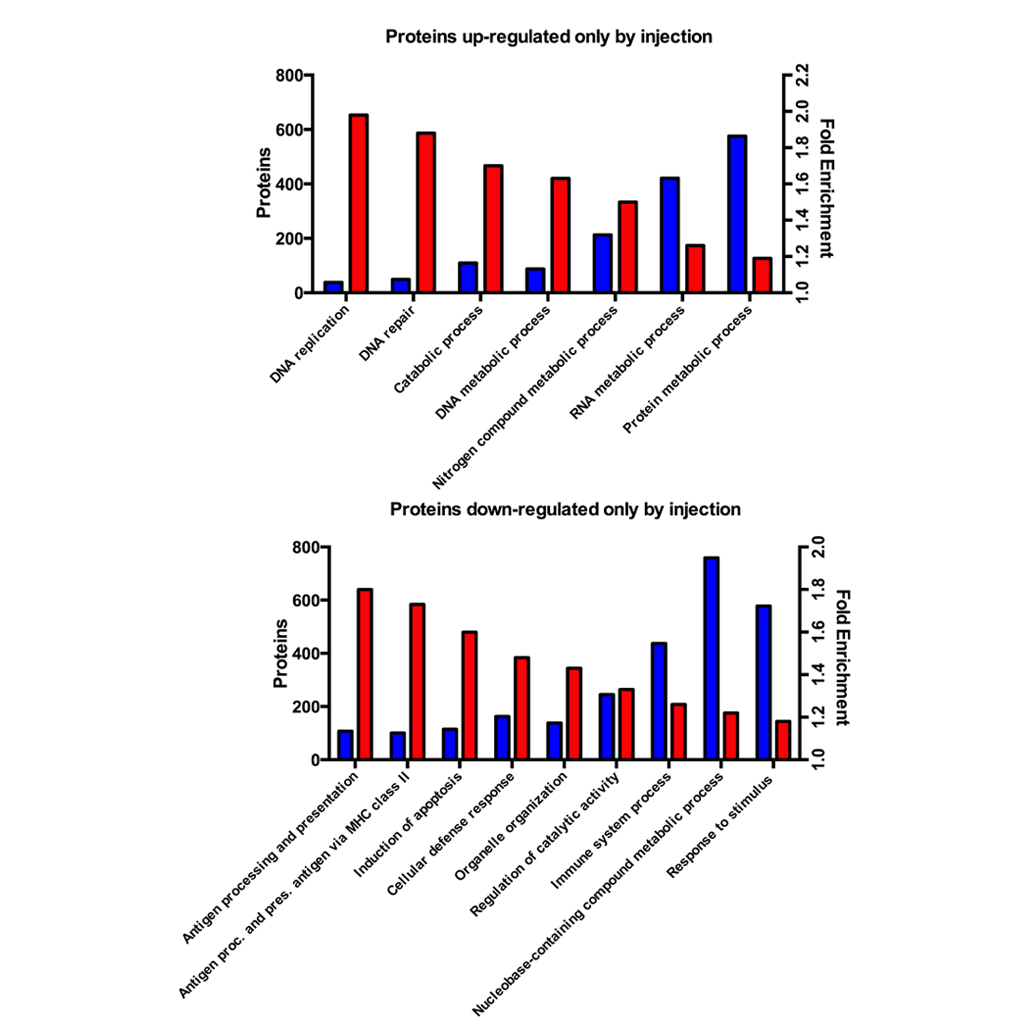

Supplement: Figure S2 — Overrepresentation analysis for proteins changed only in injection method. Up-regulated (top) or down-regulated (bottom) proteins at 22 HPI in zebrafish exposed at 72 HPF by injection (but not in larvae exposed by immersion) categorized by biological process (GO). Blue bars indicate number of proteins. Red bars indicate fold enrichment. The cut-off was set at P < 0.05. Only the more relevant groups are presented. Proc, processing. Pres, presentation. [file Image2.TIF]

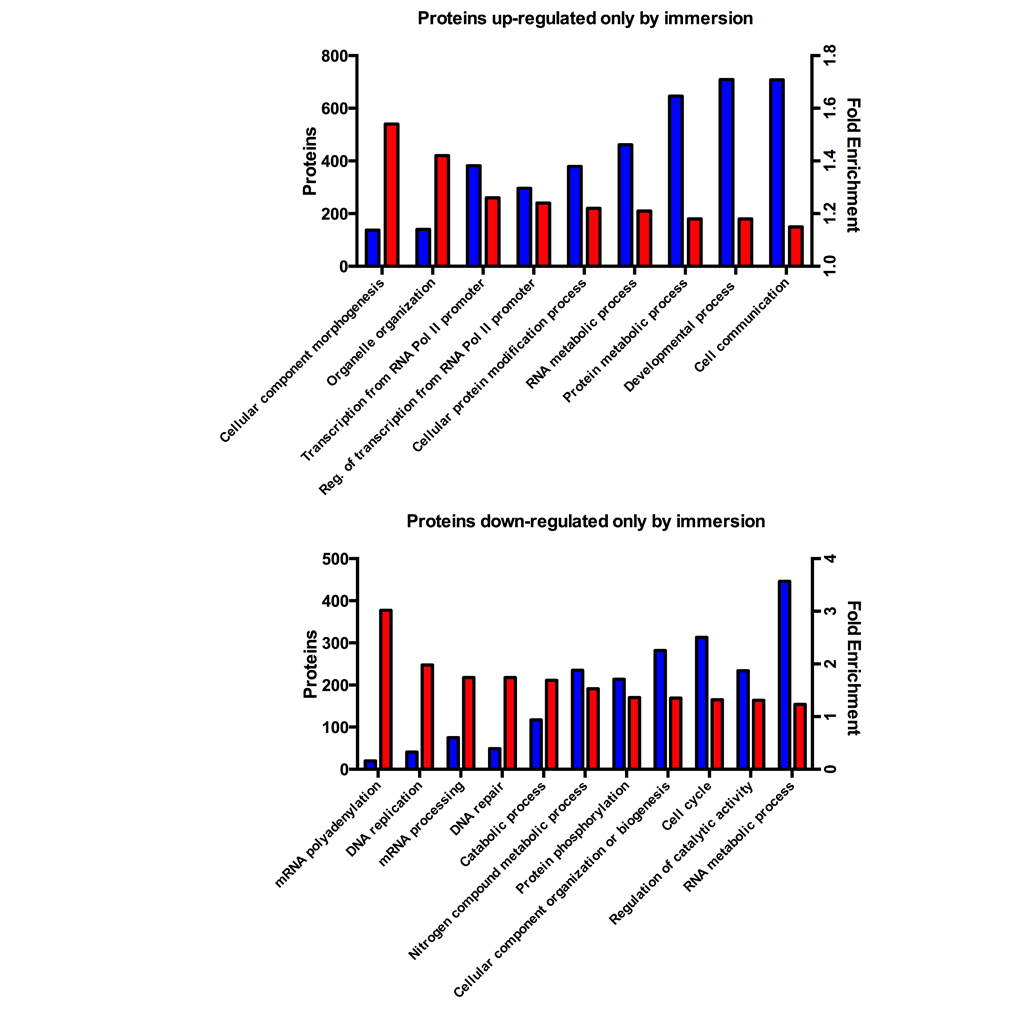

Supplement: Figure S3 — Overrepresentation analysis for proteins changed only in immersion method. Up-regulated (top) or down-regulated (bottom) proteins at 22 hpe in zebrafish exposed at 72 HPF by immersion (but not in larvae exposed by injection) categorized by biological process (GO). Blue bars indicate number of proteins. Red bars indicate fold enrichment. The cut-off was set at P < 0.05. Only the more relevant groups are presented. Reg, regulation. [file Image3.TIF]
