# Supplementary material for: In vivo Host-Pathogen Interaction as Revealed by Global Proteomic Profiling of Zebrafish Larvae
Source: Front Cell Infect Microbiol. 2017 Jul 25;7:334. doi: 10.3389/fcimb.2017.00334 (PMC5524664; doi:10.3389/fcimb.2017.00334)
Supplement: Table S1 — Overrepresentation test of proteins up-regulated and down-regulated in larvae exposed to P. aeruginosa according to Biological process. The up-regulated and down-regulated proteins in zebrafish injected with P. aeruginosa PAO1 or co-incubated with P. aeruginosa PAO1, with respect to the controls, were analyzed according to Biological Process. The cut of was set to P < 0.05. [file Table1.DOCX]

**Supplementary Table 1. Overrepresentation test of proteins up-regulated and down-regulated in larvae exposed to *P. aeruginosa* according to Biological process.** The up-regulated and down-regulated proteins in zebrafish injected with *P. aeruginosa* PAO1 or co-incubated with *P. aeruginosa* PAO1, with respect to the controls, were analysed according to Biological Process. The cut of was set to P-value<0.05

| **Biological Process** | **Proteins found** | **Expected proteins** | **Fold enrichment** | **P-value** |
| --- | --- | --- | --- | --- |
| **Proteins up-regulated and down-regulated only in injected zebrafish** | | | | |
| DNA repair | 94 | 55,06 | 1,71 | 2,25E-04 |
| DNA replication | 68 | 40,63 | 1,67 | 1,13E-02 |
| antigen processing and presentation | 181 | 112,77 | 1,61 | 3,34E-07 |
| antigen processing and presentation of peptide or polysaccharide antigen via MHC class II | 172 | 109,24 | 1,57 | 2,99E-06 |
| induction of apoptosis | 202 | 136,03 | 1,48 | 1,24E-05 |
| chromatin organization | 106 | 71,55 | 1,48 | 1,67E-02 |
| DNA metabolic process | 165 | 112,77 | 1,46 | 4,38E-04 |
| mitosis | 181 | 126,9 | 1,43 | 6,48E-04 |
| catabolic process | 185 | 135,73 | 1,36 | 6,33E-03 |
| cellular defense response | 278 | 209,34 | 1,33 | 5,42E-04 |
| organelle organization | 240 | 183,14 | 1,31 | 5,81E-03 |
| cellular component movement | 222 | 171,36 | 1,3 | 2,16E-02 |
| regulation of molecular function | 455 | 354,21 | 1,28 | 1,67E-05 |
| regulation of catalytic activity | 447 | 349,2 | 1,28 | 3,17E-05 |
| cellular component organization or biogenesis | 567 | 443,71 | 1,28 | 7,95E-07 |
| cellular component organization | 520 | 409,27 | 1,27 | 7,32E-06 |
| nucleobase-containing compound metabolic process | 1491 | 1178,63 | 1,27 | 1,01E-19 |
| cell adhesion | 299 | 242,91 | 1,23 | 4,81E-02 |
| nitrogen compound metabolic process | 367 | 300,03 | 1,22 | 1,59E-02 |
| metabolic process | 3538 | 2924,33 | 1,21 | 6,05E-45 |
| RNA metabolic process | 853 | 710,18 | 1,2 | 5,06E-06 |
| primary metabolic process | 2907 | 2430,86 | 1,2 | 6,03E-29 |
| regulation of nucleobase-containing compound metabolic process | 629 | 528,51 | 1,19 | 1,20E-03 |
| transcription from RNA polymerase II promoter | 672 | 565,32 | 1,19 | 6,51E-04 |
| transcription, DNA-dependent | 697 | 587,4 | 1,19 | 5,25E-04 |
| regulation of transcription from RNA polymerase II promoter | 524 | 446,07 | 1,17 | 2,42E-02 |
| immune system process | 762 | 656,59 | 1,16 | 2,96E-03 |
| localization | 1119 | 975,76 | 1,15 | 1,65E-04 |
| cellular process | 2806 | 2448,23 | 1,15 | 3,32E-16 |
| transport | 1065 | 934,54 | 1,14 | 8,75E-04 |
| developmental process | 1266 | 1124,45 | 1,13 | 7,64E-04 |
| protein metabolic process | 1149 | 1022,57 | 1,12 | 3,27E-03 |
| biological regulation | 1519 | 1366,47 | 1,11 | 7,73E-04 |
| cell communication | 1260 | 1145,06 | 1,1 | 3,08E-02 |
| **Proteins up-regulated and down-regulated in zebrafish exposed by immersion** | | | | |
| DNA replication | 71 | 42,54 | 1,67 | 8,58E-03 |
| DNA repair | 95 | 57,65 | 1,65 | 8,18E-04 |
| DNA metabolic process | 178 | 118,07 | 1,51 | 2,91E-05 |
| chromatin organization | 112 | 74,91 | 1,5 | 7,56E-03 |
| mitosis | 190 | 132,87 | 1,43 | 3,24E-04 |
| cellular component organization or biogenesis | 654 | 464,57 | 1,41 | 1,32E-15 |
| anatomical structure morphogenesis | 309 | 219,8 | 1,41 | 1,05E-06 |
| cellular component morphogenesis | 242 | 173,25 | 1,4 | 7,52E-05 |
| cellular component biogenesis | 141 | 101,12 | 1,39 | 2,01E-02 |
| cellular component organization | 594 | 428,51 | 1,39 | 8,12E-13 |
| catabolic process | 194 | 142,12 | 1,37 | 3,82E-03 |
| organelle organization | 261 | 191,75 | 1,36 | 1,89E-04 |
| antigen processing and presentation | 159 | 118,07 | 1,35 | 3,77E-02 |
| induction of apoptosis | 189 | 142,42 | 1,33 | 2,11E-02 |
| protein phosphorylation | 424 | 323,69 | 1,31 | 6,26E-06 |
| nitrogen compound metabolic process | 407 | 314,14 | 1,3 | 3,61E-05 |
| cellular component movement | 231 | 179,42 | 1,29 | 2,27E-02 |
| regulation of molecular function | 473 | 370,86 | 1,28 | 2,12E-05 |
| regulation of catalytic activity | 464 | 365,62 | 1,27 | 4,81E-05 |
| nucleobase-containing compound metabolic process | 1548 | 1234,04 | 1,25 | 4,62E-19 |
| phosphate-containing compound metabolic process | 523 | 418,02 | 1,25 | 4,29E-05 |
| cell cycle | 605 | 485,23 | 1,25 | 7,14E-06 |
| metabolic process | 3772 | 3061,82 | 1,23 | 1,75E-57 |
| primary metabolic process | 3117 | 2545,14 | 1,22 | 7,29E-40 |
| cellular protein modification process | 739 | 604,84 | 1,22 | 4,41E-06 |
| RNA metabolic process | 907 | 743,57 | 1,22 | 1,17E-07 |
| protein metabolic process | 1289 | 1070,65 | 1,2 | 3,16E-10 |
| protein transport | 507 | 424,81 | 1,19 | 7,87E-03 |
| transcription, DNA-dependent | 732 | 615,01 | 1,19 | 2,02E-04 |
| intracellular protein transport | 491 | 412,78 | 1,19 | 1,40E-02 |
| transcription from RNA polymerase II promoter | 700 | 591,89 | 1,18 | 7,85E-04 |
| cellular process | 3015 | 2563,33 | 1,18 | 8,35E-25 |
| regulation of nucleobase-containing compound metabolic process | 649 | 553,36 | 1,17 | 4,54E-03 |
| biological regulation | 1655 | 1430,72 | 1,16 | 1,44E-08 |
| developmental process | 1351 | 1177,31 | 1,15 | 8,28E-06 |
| localization | 1170 | 1021,63 | 1,15 | 1,22E-04 |
| transport | 1108 | 978,47 | 1,13 | 1,58E-03 |
| cell communication | 1342 | 1198,89 | 1,12 | 1,12E-03 |
| regulation of biological process | 1177 | 1064,17 | 1,11 | 2,79E-02 |
| DNA replication | 71 | 42,54 | 1,67 | 8,58E-03 |
| DNA repair | 95 | 57,65 | 1,65 | 8,18E-04 |
| DNA metabolic process | 178 | 118,07 | 1,51 | 2,91E-05 |
| chromatin organization | 112 | 74,91 | 1,5 | 7,56E-03 |
